# Impact of Preoperative Nutritional Status on Postoperative Outcomes in Laparoscopic Cholecystectomy: A Study From Basra Teaching Hospital

**DOI:** 10.7759/cureus.88357

**Published:** 2025-07-20

**Authors:** Tahseen A Hasan, Ali Jabbari, Hassan Mirzaei, Hamed A Flaifel, Somayeh Ghorbani, Omid Nikpayam, Mostafa Mohammadi

**Affiliations:** 1 Ischemic Disorders Research Center, Golestan University of Medical Sciences, Gorgan, IRN; 2 Department of Anesthesiology and Critical Care Medicine, Ischemic Disorders Research Center, Golestan University of Medical Sciences, Gorgan, IRN; 3 Department of Anesthesia Technologies, Alfarqadein University College, Basra, IRQ; 4 Cancer Research Center, Golestan University of Medical Sciences, Gorgan, IRN; 5 Department of Nutritional Sciences, School of Health, Golestan University of Medical Sciences, Gorgan, IRN; 6 Department of Anesthesiology and Intensive Care, Imam Khomeini Hospital Complex, Tehran University of Medical Sciences, Tehran, IRN

**Keywords:** los, mna-sf, nutrition status, pain, ponv

## Abstract

Background

Malnutrition is associated with adverse events, and nutritional status is a significant determinant of surgical outcomes. Its precise effect on typical postoperative outcomes after laparoscopic cholecystectomy (LC), a common short-stay procedure carried out in a population that is generally healthy, is still up for debate.

Objective

The primary objective of this investigation was to assess the relationship between the incidence of postoperative pain, postoperative nausea and vomiting (PONV), and length of hospital stay (LOS) and preoperative nutritional status as determined by the Mini Nutritional Assessment-Short Form (MNA-SF). Investigating the connections between serum albumin, body mass index (BMI), and the primary outcomes of nutritional status and kidney function was the secondary goal.

Methods

A total of 140 patients, aged 30 to 60 years, who had elective LC at Basra Teaching Hospital were included in this prospective observational study. Demographic information, laboratory markers like serum albumin, and nutritional assessments using the MNA-SF and BMI were all part of the preoperative evaluations. LOS, PONV incidence, and pain (Visual Analogue Scale) were among the postoperative outcomes that were prospectively documented over 24 hours. To evaluate correlations and associations, statistical analyses were conducted.

Results

The mean age of the 140 patients was 40.7±8.98 years, and 125 (89.3%) were female. The MNA-SF found that 26 patients (18.6%) were malnourished and 72 patients (51.4%) were at risk of malnutrition. There was no significant correlation found between the MNA-SF score and LOS (p=0.984), PONV (p=0.317), or postoperative pain (p=0.468). On the other hand, a lower incidence of PONV was substantially linked to a higher BMI (p=0.049). There was a significant correlation between lower postoperative systolic (p=0.005) and diastolic (p=0.039) blood pressure and lower preoperative serum albumin levels.

Conclusion

Although the MNA-SF's overall nutritional screening seems to have little predictive ability for common complications in this non-elderly LC cohort, certain indicators, such as serum albumin and BMI, may be able to help identify patients who are at risk for particular adverse events. These indicators may help guide focused perioperative strategies, as evidenced by the correlations found between lower albumin and a propensity for postoperative hypotension and between higher BMI and lower PONV.

## Introduction

A patient's nutritional status is a critical modulator of the physiological stress response. Within the surgical domain, malnutrition is a prevalent yet frequently underdiagnosed condition linked to a cascade of adverse events, including impaired wound healing, infectious complications, prolonged hospitalization, and increased healthcare expenditures [1]. A substantial portion of hospitalized patients, particularly those undergoing gastrointestinal surgery, exhibit some degree of malnutrition, underscoring the necessity of preoperative nutritional assessment in comprehensive patient management [2].

Laparoscopic cholecystectomy (LC) is the standard of care for symptomatic gallstone disease. Its minimally invasive nature confers significant advantages, such as reduced postoperative pain, shorter recovery periods, and lower morbidity compared to open surgery. Nevertheless, patients undergoing LC commonly experience undesirable symptoms like significant pain, postoperative nausea and vomiting (PONV), and delayed recovery, which can negatively affect patient satisfaction and resource utilization [3].

Various screening tools are employed to identify patients at higher risk for such complications. The Mini Nutritional Assessment-Short Form (MNA-SF) is a widely adopted and validated instrument for this purpose, though its development and validation have primarily centered on geriatric populations. Other common metrics include anthropometric measurements like Body Mass Index (BMI) and biochemical markers such as serum albumin, which reflects both nutritional state and systemic inflammation [4].

The link between poor nutritional status and adverse outcomes is well-documented in patients undergoing major, high-stress surgery or in frail, elderly populations. Its relevance in a younger, generally healthier cohort (ASA class I-II) undergoing a minimally invasive, short-stay procedure like LC is less certain. The physiological insult of LC may be insufficient to unmask the clinical consequences of moderate nutritional deficits [2].

This study was designed to address this knowledge gap. The primary objective was to evaluate the association between preoperative nutritional status, as assessed by the MNA-SF, and the incidence of postoperative pain, PONV, and length of hospital stay. Secondary objectives were to explore the relationships between BMI, serum albumin, and these same outcomes. We hypothesized that poorer nutritional status would be associated with a higher incidence of postoperative complications.

## Materials and methods

Methodology

Study Design and Setting

This prospective observational study was conducted from July to September 2024 at Basra Teaching Hospital in Basra, Iraq. The study protocol received approval from the Ethics Committee of the Ischemic Disease Research Center of Golestan University of Medical Sciences (IR.GOUMS.REC.1403.004). All participants provided written informed consent following a detailed explanation of the study's purpose and procedures and were informed of their right to withdraw at any time.

Patient Population and Criteria

The study enrolled 140 patients scheduled for elective LC with an American Society of Anesthesiologists (ASA) physical status of I or II. All enrolled participants completed the study without loss to follow-up. Eligibility criteria stipulated an age range of 30 to 60 years and a confirmed diagnosis of gallstones. Patients were excluded if they presented with concurrent jaundice or pancreatitis, required simultaneous surgical procedures, were undergoing open cholecystectomy, or were unable to provide consent. Exclusion criteria are detailed in Table 1.

**Table 1:** Inclusion and Exclusion Criteria for Study Participants

Criterion Type	Criterion Description
Inclusion Criteria	Patients age 30-60 years
	Diagnosed with gallstones
	Subject to Laparoscopic Cholecystectomy (LC)
	Able to communicate and willing to answer questions
Exclusion Criteria	Patients with jaundice or pancreatitis
	Patients who did not agree to participate in the study
	Patients with other health conditions requiring concurrent surgical intervention
	Patients who were unable to speak or had mental disorders
	Patients undergoing open cholecystectomy

Preoperative Assessments

A comprehensive preoperative assessment was performed for all participants, which included the collection of demographic data, medical history, and nutritional evaluations.

Nutritional screening (MNA-SF): The MNA-SF questionnaire was the primary nutritional screening instrument. It consists of six items assessing food intake, weight loss, mobility, recent psychological stress or acute illness, neuropsychological problems, and BMI Scores are categorized as follows: 12-14 points (normal nutritional status), 8-11 points (at risk of malnutrition), and 0-7 points (malnourished). The questionnaire was translated into Arabic by the certified Iraqi Translators Association to ensure semantic equivalence, though formal psychometric validation of this specific translation was not conducted as part of this study.

Anthropometric and laboratory measurements: Height and weight were measured to calculate Body Mass Index (BMI) as weight (kg) / height (m2). Patients were categorized according to WHO criteria: <18.5 (underweight), 18.5-24.9 (normal weight), 25.0-29.9 (overweight), and ≥30.0 (obese) [5]. Class III (formerly morbid) obesity, defined as a BMI ≥40 or ≥35 with significant comorbidities, was noted but not used as a separate category in the primary analysis due to insufficient sample size in this stratum [6].

Laboratory investigations: Fasting venous blood samples were collected preoperatively to measure serum albumin, hemoglobin, and other standard laboratory markers. Hypoalbuminemia was defined as a serum albumin level below 3.5 g/dl.

Anesthesia and Perioperative Management

All patients underwent a standardized general anesthetic.

Monitoring and induction: In the operating room, standard monitoring included 5-lead ECG, non-invasive blood pressure (NIBP), pulse oximetry (SpO2), end-tidal carbon dioxide (ETCO2), and heart rate (HR). Following pre-oxygenation, anesthesia was induced with intravenous propofol (1.5−2.0 mg/kg), fentanyl (1-2 mcg/kg), and rocuronium (0.6 mg/kg) to facilitate endotracheal intubation.

Maintenance and neuromuscular monitoring: Sevoflurane (0.5-3%) or isoflurane (1-2.5%) was used for maintenance anaesthesia. Intra-operative neuromuscular function was monitored with a Train-of-Four (TOF) nerve stimulator positioned over the ulnar nerve. We tried to maintain a deep level of neuromuscular blockade (TOF count 0-1) to provide optimal surgical conditions.

Mechanical ventilation: Mechanical ventilation was carried out on patients through a volume-controlled ventilation (VCV) mode. Tidal volume (TV) was initially adjusted between 6-8 mL/kg of predicted body weight, respiratory rate between 12-16 breaths/min to maintain the ETCO2 between 35-45 mmHg, and Positive End-Expiratory Pressure (PEEP) was adjusted to 5 cmH2O to prevent atelectasis.

Fluid management: The intraoperative fluid management included a bolus and maintenance infusion of lactated Ringer's solution 4-6 mL/kg/hr, titrated to hemodynamic parameters to ensure euvolemia and avoid hypovolemia-induced PONV.

Reversal and extubation: After surgery, residual neuromuscular block was reversed with neostigmine (0.05 mg/kg) and glycopyrrolate (0.01 mg/kg). Glycopyrrolate was used instead of atropine because it cannot cross the blood-brain barrier, a characteristic that can minimize central anticholinergic side effects without impairing equivalent vagolysis [2]. Reversal was delayed until a TOF count of 4 was obtained. Patients were extubated when they fulfilled standard criteria, which included satisfactory spontaneous respiration and hemodynamic stability [2,4].

Recovery: Ongoing monitoring of vital signs during the recovery unit and supplementary oxygen via mask for about 30 minutes before discharge to the ward.

Postoperative Outcome Measurement

Postoperative patients were monitored in the surgical ward for 24 hours. Prospectively documented and analyzed end points in this study included postoperative pain, PONV, and LOS. Additional routine surgical complications (e.g., bleeding, conversion to an open procedure, surgical site infection) were monitored, but none of these complications took place in our study population.

Postoperative pain management and assessment: Postoperative pain was treated with a standard multimodal regimen based on PROSPECT guidelines. All patients received regularly scheduled oral paracetamol and an NSAID (e.g., ibuprofen) for the initial 24 hours, unless contraindicated. The intensity of pain was evaluated on the 10-point Visual Analogue Scale (VAS), from no pain (0) to worst possible pain (10). VAS score 1-4 was mild, 5-6 moderate, and 7-10 severe pain. Rescue analgesia (VAS score > 4) was provided with intravenous fentanyl 25-50 mcg to patients [3,7].

PONV assessment and treatment: Prophylactic antiemetics were given according to Apfel score risk stratification. All patients received intravenous dexamethasone (4-8 mg) after induction. The incidence of nausea or vomiting was noted as episodes during the initial 24 hours after surgery. Treatment for established PONV (any episode of vomiting or patient request for treatment) was intravenous ondansetron (4 mg) [8].

Length of stay (LOS) in hospital: LOS was quantified in days, from the surgery day to the discharge day, based on the patient's official documents of discharge [9].

Blood pressure: Systolic and diastolic blood pressure was recorded as continuous variables postoperatively in the recovery room. For reference, hypotension was considered systolic <90 mmHg or diastolic <60 mmHg, but analyses were conducted on continuous measures.

Surgical Technique

All surgeries were performed by experienced surgeons using a standard four-port laparoscopic technique. Pneumoperitoneum was established with CO2 insufflation, maintaining an intra-abdominal pressure of 12 mmHg. The cystic artery and duct were ligated with clips before the gallbladder was excised [10].

Statistical Analysis

Data were analyzed using IBM SPSS statistical software Version 26.0 (IBM Corp., Armonk, NY, USA). The normality of continuous data was assessed with the Kolmogorov-Smirnov test. Categorical variables are presented as frequencies and percentages (N, %), while continuous variables are presented as Mean ± Standard Deviation (SD).

The association between categorical preoperative variables (e.g., MNA-SF categories, BMI categories) and categorical postoperative outcomes (e.g., PONV incidence) was assessed using the Chi-square (X2) test.

A one-way Analysis of Variance (ANOVA) was used to compare the means of continuous variables (e.g., BMI, albumin) across the three MNA-SF nutritional groups. For comparisons between two groups, the independent samples Student's t-test was used [11].

Pearson's correlation coefficient (r) was utilized to determine if there was a relationship between two continuous variables, i.e., between preoperative serum albumin and postoperative systolic blood pressure (SBP) and diastolic blood pressure (DBP). Statistically significant was kept at 0.05 for all tests. Association here is used to refer to associations between categorical variables (chi-square tests), and a relationship between continuous variables is described using correlation (Pearson’s correlation coefficient).

## Results

The study included 140 participants who underwent elective laparoscopic cholecystectomy. The demographic and baseline clinical characteristics are presented in Table 2. The cohort was predominantly female (125 patients, 89.3%), with a mean age of 40.70 ± 8.98 years. The nutritional status results based on MNA-SF showed that 72 patients (51.4%) were at risk of malnutrition and 26 (18.6%) were malnourished. In terms of education, 94 patients (67.1%) were categorized as illiterate or having completed only primary education. Anthropometric analysis revealed that 69 patients (49.3%) were obese (BMI ≥30.0), and an additional 46 (32.9%) were overweight. Based on serum albumin levels, 25 patients (17.85%) were classified as malnourished (albumin <3.5 g/dl) (Table 2).

**Table 2:** Baseline characteristics of patients MNA-SF: Mini Nutritional Assessment-Short Form; BMI: Body Mass Index; SBP: Systolic Blood Pressure; DBP: Diastolic Blood Pressure; HR: Heart Rate; Mg: Magnesium; Chol: Cholesterol; Tg: Triglycerides; HB: Hemoglobin; RBC: Red Blood Cell; SD: Standard Deviation; n: frequency; %: percentage.

Variables		Mean±SD or N (%)
Age (years)		40.70±8.98
Sex	Male	15 (10.7%)
	Female	125 (89.3%)
BMI (kg/m^2^)	Underweight < 18.5	3 (2.1%)
	Normal = 18.5-24.9	22 (15.7%)
	Overweight = 25-29.99	46 (32.9%)
	Obese > 30	69 (49.3%)
MNA	Normal nutrition	42 (30.0%)
	Risk of malnutrition	72 (51.4%)
	Malnutrition	26 (18.6%)
Educational level	Illiterate or primary	94 (67.1%)
	Secondary	34 (24.3%)
	Academic	12 (8.6%)
Hypertension	No	111 (79.3%)
	Yes	29 (20.7%)
Surgery history	No	81 (58.3%)
	One	29 (20.3%)
	More	29 (20.3%)
Treatment history	No	74 (52.9%)
	One	50 (35.7%)
	More	16 (11.4%)
Preoperative SBP (mmHg)		124.84±13.44
Preoperative DBP (mmHg)		80.49±9.21
Preoperative HR (beats per min)		86.11±12.21
Contraceptives	No	110 (78.6%)
	Yes	30 (21.4%)
Co-morbid diseases	No	73 (52.1%)
	One	51 (36.4%)
	More	16 (11.4%)
Albumin (g/dl)	Malnourished <3.5	25 (17.85%)
	Well-nourished ≥ 3.5	115 (82.14%)
Mg (mg/dl)		1.70 ± .26
Chol (mg/dl)		159.18 ± 46.56
Tg (mg/dl)		128.10 ± 68.41
HB (g/dl)		11.84 ± 1.47
RBC (million cells/mcl)		4.69 ± .52

Postoperative complications results showed that 33 (23.6%) of patients experienced one-time PONV, and 21 (15%) with two or more episodes; 54 (38.6%) of patients had severe pain, while 36 (25.7%) of them had moderate pain, as shown in Table 3. The mean LOS was 1.47 ± 1.24 days. Blood pressure measurements were taken at a single time point in the recovery room.

**Table 3:** Postoperative variables PONV: Postoperative Nausea and Vomiting; SBP: Systolic Blood Pressure; DBP: Diastolic Blood Pressure; HR: Heart Rate; SD: Standard Deviation; n: frequency; %: percentage

Variables		Mean ± SD or N (%)
PONV	No	86 (61.4%)
	One time	33 (23.6%)
	Two or more	21 (15.0%)
Postoperative pain	Mild	50 (35.7%)
	Moderate	36 (25.7%)
	Severe	54 (38.6%)
Length of stay in hospital (days)		1.47 ± 1.24
Postoperative SBP (mmHg)		124.52 ± 13.65
Postoperative DBP (mmHg)		75.94 ± 9.51
Postoperative HR (beats per min)		80.07 ± 8.73

In comparison, patients’ baseline characteristics with nutritional status were based on MNA-SF. It was observed that BMI, albumin, magnesium (Mg), and cholesterol were associated with nutritional status based on MNA-SF significantly (p=0.001, p <0.001, p=0.019, p=0.021, respectively). Furthermore, the study showed that albumin was significantly correlated with MNA-SF (p< 0.001). All other variables had no significant effect on MNA-SF, as shown in Table 4.

**Table 4:** Comparison of baseline characteristics with nutritional status based on MNA-SF. -: Assumptions of the test were not met. *: One-way ANOVA #: Chi-Square test MNA-SF: Mini Nutritional Assessment-Short Form; BMI: Body Mass Index; SBP: Systolic Blood Pressure; DBP: Diastolic Blood Pressure; HR: Heart Rate; Mg: Magnesium; Chol: Cholesterol; TG: Triglycerides; HB: Hemoglobin; RBC: Red Blood Cell; SD: Standard Deviation; n: frequency; %: percentage; P-value: probability value; df: degrees of freedom.

Characteristics		MNA-SF			Test value^*^	df	P-value
		Normal nutrition (n=42)	At risk of malnutrition (n=72)	Malnutrition (n=26)			
Sex n (%)	Male	3 (20.0)	7 (46.7)	5 (33.3)	-^#^	-	-
	Female	39 (31.2)	65 (52.0)	21 (16.8)			
Educational level n (%)	Illiterate or primary	28 (29.8)	50 (53.2)	16 (17.0)	3.399^#^	4	0.493
	Secondary	11 (32.4)	14 (41.2)	9 (26.5)			
	Academic	3 (25.0)	8 (66.7)	1 (8.3)			
Hypertension n (%)	No	32 (28.8)	57 (51.4)	22 (19.8)	0.695^#^	2	0.706
	Yes	10 (34.5)	15 (51.7)	4 (13.8)			
Contraceptives n (%)	No	32 (29.1)	54 (49.1)	24 (21.8)	3.601^#^	2	0.165
	Yes	10 (33.3)	18 (60.0)	2 (6.7)			
Surgery history n (%)	No	28 (34.6)	37 (45.7)	16 (19.8)	4.827^#^	4	0.306
	One	5 (17.2)	17 (58.6)	7 (24.1)			
	More	9 (31.0)	17 (58.6)	3 (10.3)			
Treatment history n (%)	No	23 (31.1)	41 (55.4)	10 (13.5)	2.851^#^	4	0.583
	One	15 (30.0)	23 (46.0)	12 (24.0)			
	More	4 (25.0)	8 (50.0)	4 (25.0)			
Comorbidity diseases n (%)	No	22 (30.1)	41 (56.2)	10 (13.7)	2.985^#^	4	0.560
	One	15 (29.4)	23 (45.1)	13 (25.5)			
	More	5 (31.3)	8 (50.0)	3 (18.8)			
Age (years)	Mean ± SD	41.48 ± 7.45	40.44 ± 9.67	40.15 ± 9.48	0.232	2	0.793
BMI (kg/m^2^)	Mean ± SD	32.10 ± 5.80	28.74 ± 4.96	27.12 ± 7.18	7.322	2	0.001
SBP (mmHg)	Mean ± SD	122.57 ± 13.28	125.14 ± 11.59	127.65 ± 17.77	1.190	2	0.307
DBP (mmHg)	Mean ± SD	80.62 ± 8.88	79.58 ± 8.34	82.77 ± 11.72	1.151	2	0.319
HR (beats per minute)	Mean ± SD	87.67 ± 11.75	84.19 ± 11.76	88.92 ± 13.64	1.945	2	0.147
Albumin (g/dl)	Mean ± SD	4.35 ± 0.81	4.09 ± 0.57	3.63 ± 0.84	8.247	2	<0.001
Mg (mg/dl)	Mean ± SD	1.78 ± 0.29	1.68 ± 0.25	1.61 ± 0.22	4.092	2	0.019
Chol (mg/dl)	Mean ± SD	175.47 ± 49.55	150.81 ± 41.70	156.06 ± 49.29	3.955	2	0.021
TG (mg/dl)	Mean ± SD	134.40 ± 44.32	124.72 ± 81.82	127.30 ± 60.69	0.264	2	0.768
HB (g/dl)	Mean ± SD	11.90 ± 1.09	11.71 ± 1.39	12.08 ± 2.11	0.669	2	0.514
RBC (million cells/mcl)	Mean ± SD	4.61 ± 0.46	4.73 ± 0.48	4.69 ± 0.70	0.736	2	0.481

**Figure 1:**
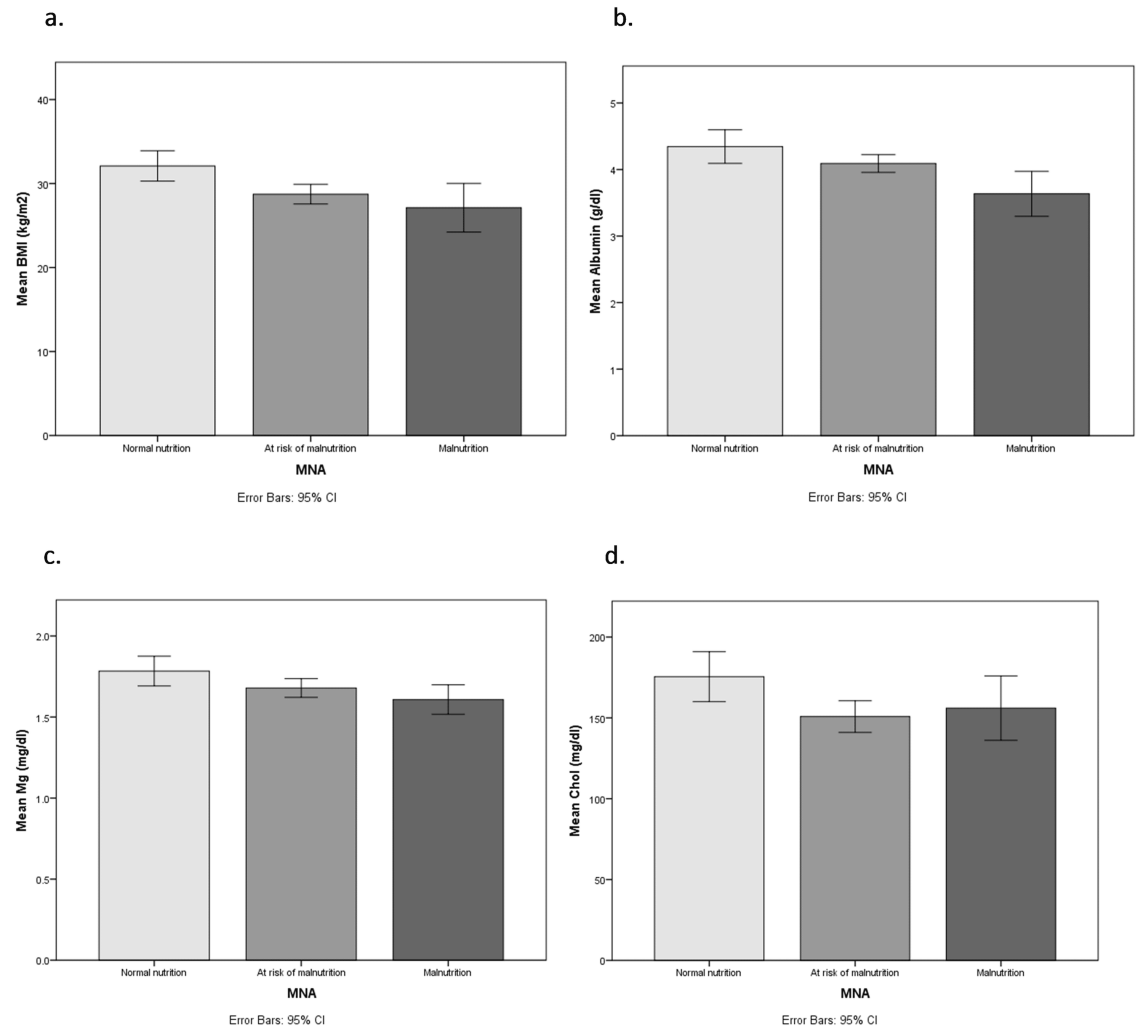
Relationship between preoperative nutritional status based on MNA-SF with (a) BMI, (b) Albumin, (c) Magnesium, and (d) Cholesterol MNA-SF: Mini Nutritional Assessment-Short Form

Our findings showed that patients’ nutritional status based on their MNA-SF had a significant correlation with BMI, albumin, magnesium (mg), and cholesterol (Figure 1).

Results showed no correlation between nutritional status based on MNA-SF and postoperative complications, PONV, pain, and length of hospital stay (p = 0.317, 0.468, and 0.984), respectively (Table 5).

**Table 5:** Comparison of postoperative outcomes with nutritional status based on MNA-SF. *: One-way ANOVA #: Chi-Square test MNA-SF: Mini Nutritional Assessment-Short Form; PONV: Postoperative nausea and vomiting; SBP: Systolic blood pressure; DBP: Diastolic blood pressure; HR: Heart rate; df: degrees of freedom.

Characteristic		MNA n (%)			Test value^*^	df	P-value
		Normal nutrition (n=42)	At risk of malnutrition (n=72)	Malnutrition (n=26)			
PONV	No	30 (71.4)	44 (61.1)	12 (46.2)	4.721^#^	4	0.317
	One	8 (19.0)	16 (22.2)	9 (34.6)			
	More	4 (9.5)	12 (16.7)	5 (19.2)			
Postoperative pain	Mild	17 (40.5)	27 (37.5)	6 (23.1)	3.565^#^	4	0.468
	Moderate	11 (26.2)	19 (26.4)	6 (23.1)			
	Severe	14 (33.3)	26 (36.1)	14 (53.8)			
Length of stay in hospital (days)	Mean ± SD	1.50 ± 1.31	1.46 ± 1.28	1.46 ± 1.07	0.016	2	0.984
Postoperative SBP (mmHg)	Mean ± SD	127.12 ± 15.57	124.13 ± 12.70	121.42 ± 12.61	1.470	2	0.234
Postoperative DBP (mmHg)	Mean ± SD	76.67 ± 9.57	75.89 ± 9.60	74.92 ± 9.46	0.269	2	0.764
Postoperative HR (beats per minute)	Mean ± SD	81.69 ± 7.28	79.40 ± 9.19	79.31 ± 9.53	1.033	2	0.359
Duration of operation (minutes)	Mean ± SD	67.14 ± 6.36	68.40 ± 7.82	68.85 ± 8.04	0.536	2	0.586

In nutritional status based on preoperative albumin, our findings found no correlation between albumin and postoperative complications (pain, PONV, and length of stay) except blood pressure (systolic and diastolic) (p=0.005, 0.039) (Table 6).

**Table 6:** Comparison of postoperative outcomes with nutritional status based on albumin. *: T-Test #: Chi-Square PONV: Postoperative nausea and vomiting; SBP: Systolic blood pressure; DBP: Diastolic blood pressure; HR: Heart rate; df: degrees of freedom.

Characteristic		Albumin n (%)		Test value^*^	df	P-value
		Malnutrition (n=25)	Healthy nourished (n=115)			
PONV	No	15 (17.4)	71 (82.6)	0.033^#^	2	0.984
	One	6 (18.2)	27 (81.8)			
	More	4 (19.0)	17 (81.0)			
Postoperative pain	Mild	10 (20.0)	40 (80.0)	0.243^#^	2	0.885
	Moderate	6 (16.7)	30 (83.3)			
	Severe	9 (16.7)	45 (83.3)			
Length of stay in hospital (days)	Mean ± SD	1.48 ± 0.92	1.47 ± 1.31	0.038	138	0.970
Postoperative SBP (mmHg)	Mean ± SD	119.40 ± 8.36	125.63 ± 14.34	-2.913	59.428	0.005
Postoperative DBP (mmHg)	Mean ± SD	72.40 ± 9.18	76.71 ± 9.45	-2.079	138	0.039
Postoperative HR (beats per minute)	Mean ± SD	79.92 ± 8.91	80.10 ± 8.73	-0.095	138	0.924
Duration of operation (minutes)	Mean ± SD	67.60 ± 8.55	68.22 ± 7.20	-0.375	138	0.708

Our study examined BMI and PONV and found a negative correlation between them (p=0.049) as shown in Table 7. Pearson correlation analysis showed that serum albumin was significantly inversely correlated with postoperative systolic BP (r = -0.24, p = 0.005) and diastolic BP (r = -0.19, p = 0.039).

**Table 7:** Relation between PONV and BMI *: T-Test #: Chi-Square PONV: Postoperative nausea and vomiting; df: degrees of freedom

Characteristic		PONV			Test value^*^	df	P-value
		No	One	More			
		Count (%)	Count (%)	Count (%)			
BMI category	Normal weight	9 (36.0)	8 (32.0)	8 (32.0)	12.667^#^	6	0.049
	Overweight	30 (65.2)	13 (28.3)	3 (6.5)			
	Class 1 Obesity	30 (68.2)	8 (18.2)	6 (13.6)			
	Class 2 Obesity	17 (68.0)	4 (16.0)	4 (16.0)			

## Discussion

Nutritional status is a very crucial aspect in surgical patients, and malnutrition is responsible for many unwanted adverse biomedical outcomes [12]. The pre-surgical nutritional assessment is therefore looked upon as being amongst the surgical patients’ outcomes improvement keys [13].

It should be remembered that there is no foolproof method, and the use of more than one method reduces the risk of errors associated with the use of any single method alone [14]. Variation in nutritional indices used in the present study ensures the maximum likelihood of screening for nutritional risk. With the use of the MNA questionnaire, 26 (18.6%) and 72 (51.4%) patients were found malnourished or at risk of malnutrition. While both the Mini Nutritional Assessment (MNA) and its short form (MNA-SF) are commonly used to assess nutritional status in geriatric patients, our study demonstrates that MNA-SF can also be effectively used as a screening tool for malnutrition in younger and middle-aged populations, a finding less investigated in previous research [15]. Gonen et al. conducted a study on 387 patients (≥18 years or older), of whom 24% were at risk of malnutrition as per MNA-SF. Besides, the study compared both MNA-SF and MNA and evaluated both of these tools in assessing nutritional status in the young and middle-aged population. There was a correlation between them, and MNA-SF was more sensitive in determining vulnerable and malnourished patients as compared to MNA [15]. Asiimwe et al. determined nutritional status through MNA-SF in subjects ≥18 years old. The prevalence of malnutrition as per MNA-SF was 59% and it is linked with BMI and serum albumin [6].

According to BMI measurement, 115 (82.2%) of our patients were overweight and obese. Furthermore, BMI is also commonly used as a good indicator of chronic energy deficiency in adults, especially in developing countries [16].

The number of female patients in our research was 125 (89.3%), and the mean age was 40.70 ± 8.98, so the female-to-male ratio is about 9 to 1. This ratio also concurs with the results of a number of studies published worldwide [12]. The most common gallbladder disease is gallstones, especially among middle-aged women [17]. Various studies suggested that the risk of gallstones is higher among women than men. The female-specific disease is supposed to be caused by hormones such as estrogen and progesterone. Estrogen causes supersaturation of cholesterol by inducing the secretion of cholesterol, and progesterone prevents gallbladder contraction and also induces cholestasis [17].

Based on all tools and assessments, the prevalence of malnutrition based on MNA-SF showed 72 (51.4%) patients at risk of malnutrition and 26 (18.6%) patients with malnutrition. Additionally, based on BMI, three (2.1%) patients were underweight, while 69 (49.3%) were obese. The study referred to all the conditions that may interfere directly or indirectly with nutritional status. These conditions include malnutrition, at-risk nutrition, underweight, overweight, obesity, anemia, and mineral deficiency.

Our findings revealed no statistical correlation between nutrition status and 24-hour postoperative pain based on all categories of tools and nutrition assessment trials. Contrarily, Chabowski et al. used the MNA questionnaire to evaluate nutritional status based on emotional status and pain and revealed that a correlation between nutritional status and pain in lung cancer patients exists [7]. Similar to our study, Kinugasa et al. evaluated nutrition based on MNA-SF and revealed that nutritional status did not have any impact on pain, nausea, and vomiting [18].

Several pieces of literature testify to the strong correlation between nutritional status and pain, in which poor nutritional status can influence mechanisms to enhance pain. Poor nutritional status can enhance the response to surgery through inflammation. High inflammation can cause increased pain perception [7, 19].

Pain after laparoscopic cholecystectomy (LC) differs from that after open surgery. This is attributed to various reasons like a quick distension of the peritoneum that results in vascular injury, traumatic traction of nerves, trauma to the abdominal wall resulting from port placement and cholecystectomy, and carbon dioxide pneumoperitoneum to attain high abdominal pressure. Incisional pain has been reported to be more intense compared to visceral pain and is most severe during the first 48 hours after laparoscopic cholecystectomy [20]. Still, regardless of whether the pain was incisional or visceral, or shoulder pain, our study showed no correlation between nutrition status and pain.

For PONV, in the current study, it is not found to be related to nutrition status based on all parameters except with BMI and triglycerides, which are related to PONV. The findings indicated that single PONV was more common in overweight patients, 13 (28.3%), but multiple PONV were more common in normal-weight patients, 8 (32.0%). This finding is in agreement with a Korean study by Kim et al. and Wei et al. that indicated that in overweight and obese patients, a reduction in PONV is observed as compared to patients with a normal BMI [21, 22]. However, many have reported that BMI and obesity as a variable have an impact on clinical outcomes like PONV, illustrating that a BMI < 24 kg/m^2^ is implicated in increased risk of PONV [23]. Contrarily, Kim et al. found that more PONV is observed in obese patients as compared to normal-weight patients due to increased fat stores with which to become saturated [21]. However, there are various risk factors that may lead to PONV, such as anesthesia, patient factors, and factors related to surgery. In addition to this, the type of surgery is a variable towards the presentation of PONV. Post laparoscopic cholecystectomy, for this reason, peritoneal distension occurs fast; there is activation of neurogenic pathways through reflex response, and through increased visceral pressure and manipulation. Formation of pneumoperitoneum is a major component of laparoscopy, leading to stretched mechanoreceptors, increased serotonin (5HT) production, and thus leading to PONV [8, 23].

There was a lack of correlation between nutritional status and postop pain, PONV, or LOS, as opposed to Chabowski et al., reporting that malnutrition correlates with pain in patients who are going through lung cancer surgery [7]. Increased BMI, though, was related to reduced risk of PONV, as reported by Kim et al. and Wei et al. [21,22]. Fentanyl dosing in VAS >4 is a risk factor, and since it has a known risk profile, it may be a risk factor for PONV as well [8]. Fluid maintenance, with risk of hypovolemia or hypervolemia, is a risk factor for PONV [9, 10]. Albumin decreased below the expected range and correlated with reduced postop systemic and diastolic blood pressure, measured at a single time-point in the recovery room, representing a hemodynamic stability role [24, 25]. While not a representative indicator of clinical hypotension (the cut-off level not specifically determined), such correlation implicates albumin as a potential marker, whereas a clear correlation with intraop and postop outcome remains to be determined [25].

The lack of an important relationship between nutritional status and postoperative pain could mirror the comparatively young, healthy (ASA I-II) group in our analysis, as small nutritional differences may have limited impact in this setting. Likewise, though we found a negative correlation between BMI and PONV (lower BMI with greater PONV), this result contrasts with some earlier reports; the difference may be explained by our sample's characteristics (mainly overweight/obese) or unmeasured variables. In addition, this weak relationship between nutritional status and pain could be explained by our overall healthy, nonelderly patient group, where modest nutritional deficits have little effect. Likewise, our result of a lower BMI correlated with greater PONV differs from some reports; this difference may be explained by study population differences or research methodology.

Strengths and limitations

Strengths

The study’s prospective design, use of multiple nutritional assessment tools (MNA-SF, BMI, albumin), and standardized perioperative protocols enhance its reliability.

Limitations

This study has a number of limitations that need to be considered when drawing inferences from results. The single-centre setting of Basra Teaching Hospital and a 140-patient sample may restrict the generalizability of results and statistical power to detect smaller effect sizes. The patient group was predominantly overweight or obese (82.2%), and this restricts conclusions about the effect of undernutrition. The Arabic Mini Nutritional Assessment-Short Form (MNA-SF) had not been formally validated, and this could undermine the reliability of determinations of nutritional status. Postoperative outcome measures such as pain (by the Visual Analogue Scale) and postoperative nausea and vomiting (PONV, by patient/staff reports) were subjectively measured, and this may introduce variability and bias. The 24-hour postoperative follow-up may not accurately reflect longer-term recovery trends or delayed complications. Unmeasured confounders such as postoperative fluid status or intraoperative opioid dose may affect outcomes.

## Conclusions

This study highlighted a significant gender difference in the prevalence of gallstone disease, with a higher proportion of female patients undergoing laparoscopic cholecystectomy. Furthermore, it was observed that patients undergoing this procedure often presented with abnormal nutritional conditions, suggesting that malnutrition is a common concern in this group. Despite this, our findings revealed no significant correlation between preoperative nutritional status and postoperative complications, such as pain, postoperative nausea and vomiting (PONV), or the length of hospital stay (LOS). This contrasts with some other studies that have reported a negative association between body mass index (BMI) and PONV, highlighting the need for further investigation into the influence of BMI on postoperative outcomes.

An interesting finding from our study was the significant relationship between preoperative albumin levels and postoperative blood pressure regulation. Albumin appears to be a valuable predictor of postoperative hemodynamic stability, which could inform clinical management strategies. This underscores the importance of monitoring nutritional markers, such as albumin, before surgery, particularly in patients with nutritional risks, to improve postoperative recovery and outcomes. While no direct association emerged between global nutritional status and postoperative complications, lower BMI correlated with higher PONV risk. Preoperative albumin levels showed an association with postoperative blood pressure changes, suggesting its potential role as a hemodynamic monitoring marker. These findings underscore the need for targeted nutritional strategies in high-risk subgroups.
